# Quantitative determination of pulmonary emphysema in follow-up LD-CTs of patients with COVID-19 infection

**DOI:** 10.1371/journal.pone.0263261

**Published:** 2022-02-03

**Authors:** Erkan Celik, Christian Nelles, Jonathan Kottlors, Philipp Fervers, Lukas Goertz, Daniel Pinto dos Santos, Tobias Achenbach, David Maintz, Thorsten Persigehl

**Affiliations:** 1 Faculty of Medicine and University Hospital Cologne, Department of Diagnostic and Interventional Radiology, University of Cologne, Cologne, Germany; 2 Department of Diagnostic and Interventional Radiology, Lahn-Dill-Kliniken, Wetzlar, Germany; Clinic for Infectious and tropical diseases, Clinical centre of Serbia, SERBIA

## Abstract

**Purpose:**

To evaluate the association between the coronavirus disease 2019 (COVID-19) and post-inflammatory emphysematous lung alterations on follow-up low-dose CT scans.

**Methods:**

Consecutive patients with proven COVID-19 infection and a follow-up CT were retrospectively reviewed. The severity of pulmonary involvement was classified as mild, moderate and severe. Total lung volume, emphysema volume and the ratio of emphysema/-to-lung volume were quantified semi-automatically and compared inter-individually between initial and follow-up CT and to a control group of healthy, age- and sex-matched patients. Lung density was further assessed by drawing circular regions of interest (ROIs) into non-affected regions of the upper lobes.

**Results:**

A total of 32 individuals (mean age: 64 ± 13 years, 12 females) with at least one follow-up CT (mean: 52 ± 66 days, range: 5–259) were included. In the overall cohort, total lung volume, emphysema volume and the ratio of lung-to-emphysema volume did not differ significantly between the initial and follow-up scans. In the subgroup of COVID-19 patients with > 30 days of follow-up, the emphysema volume was significantly larger as compared to the subgroup with a follow-up < 30 days (p = 0.045). Manually measured single ROIs generally yielded lower attenuation values prior to COVID-19 pneumonia, but the difference was not significant between groups (all p > 0.05).

**Conclusion:**

COVID-19 patients with a follow-up CT >30 days showed significant emphysematous lung alterations. These findings may help to explain the long-term effect of COVID-19 on pulmonary function and warrant validation by further studies.

## 1. Introduction

It has been more than two years, since the novel coronavirus disease 2019 (COVID-19) pandemic has begun in Wuhan, Hubei Province of China [1]. Real-time reverse-transcription polymerase chain reaction (RT-PCR), rapid antigen test, and unenhanced low-dose computed tomography of the chest (LD-CT) have emerged as the most important diagnostic tools for this disease [2]. The typical pulmonary CT manifestations of COVID-19 include bilateral ground glass opacities (GGO), consolidations located peripherally and posteriorly and crazy-paving patterns [3]. Throughout the pandemic, LD-CT was increasingly recommended for the diagnosis of COVID-19 pneumonia, since it allows to quantify pulmonary involvement and to determine the individual stage and severity of the disease [4]. During follow-up, LD-CT is employed to evaluate long-term lung parenchyma alterations such as residual ground-glass opacification, interstitial thickening and fibrotic-like lung changes [5–7]. Anatomical studies revealed that COVID-19 infection is a heterogenous disease that involves the tracheobronchial system and causes diffuse alveolar and vascular damage [8,9]. In this context, chronic respiratory diseases have been identified as predisposing risk factors for COVID-19 infection and serious adverse outcomes [10,11]. For instance, chronic obstructive pulmonary disease (COPD) correlated positively with all-cause mortality in COVID-19 patients [11,12]. As lung emphysema is caused by alveolar damaging and is involved in COPD, we evaluated, whether COVID-19 patients developed pulmonary emphysema at short-term follow-up. For this purpose, the total lung and emphysema volume was assessed semi-automatically at initial and follow-up LD-CT scans and the lung density was measured manually by drawing region of interests (ROIs) in the upper pulmonary lobes.

## 2. Material and methods

All CT-scans in this study were performed for clinical suspicion of COVID-19 infection. All data were fully anonymized before accessing. The local ethics committee approved this retrospective, single-center study (reference number 21–1216). Due to the retrospective nature of the study, a written informed consent was waived.

### 2.1 Patient population and examination selection

A database query of the institutional picture archiving and communication system (PACS) was performed searching for any patients who received a dedicated LD-CT of the chest between march 2020 and march 2021 to rule out COVID-19.

Inclusion criteria were:

1. a positive RT-PCR test,
2. an initial CT scan within 14 days after symptom onset,
3. at least one follow-up LD-CT scan.

**Fig 1:**
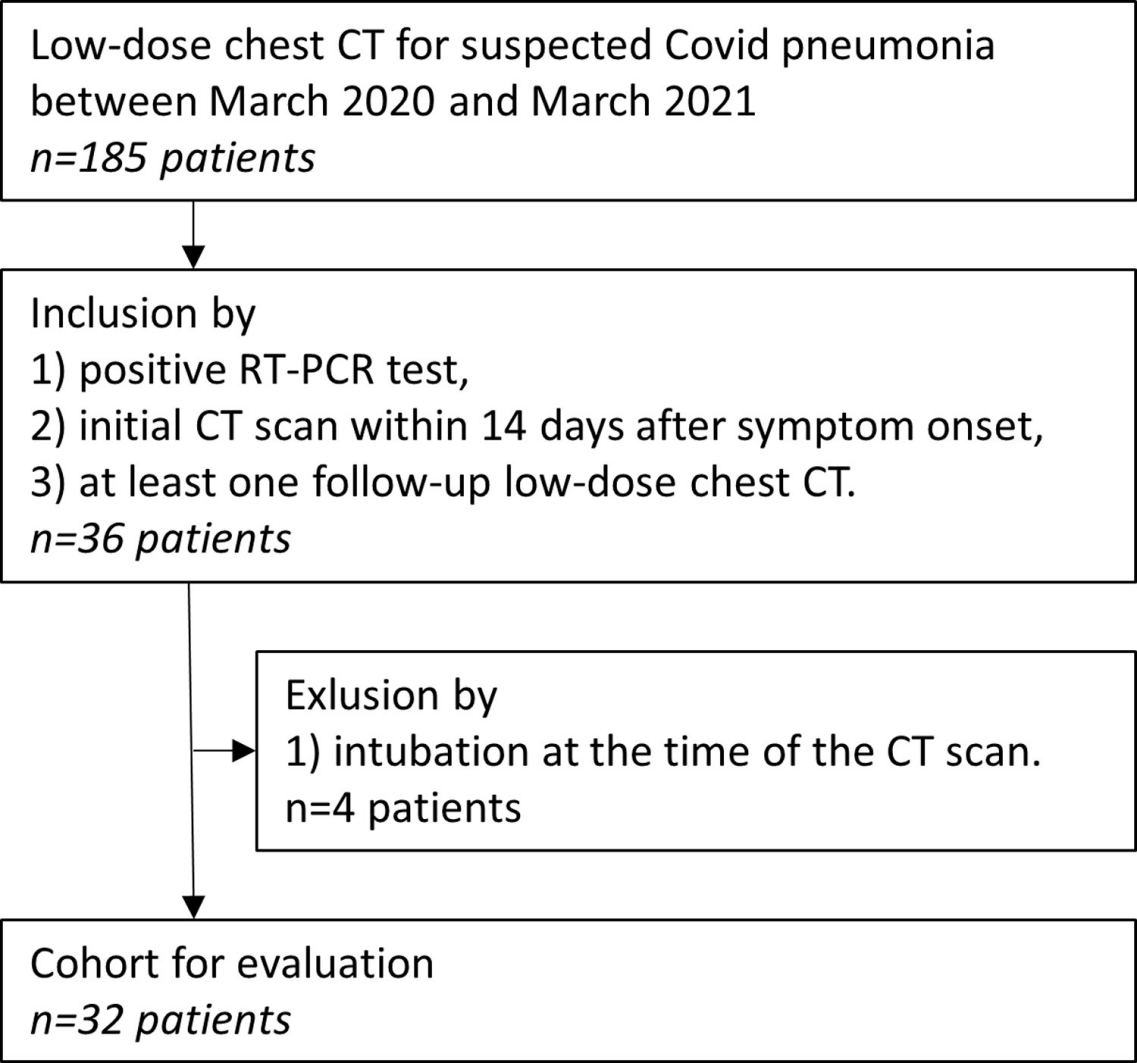
Inclusion chart of patients.

Intubated patients (n = 4) were excluded. An inclusion chart of patients and target lesions is provided in Fig 1.

LD-CT scans of five age- and sex- matched subjects with a negative RT-PCR test, no known lung diseases and at least one follow-up LD-CT scan served as the reference standard. These patients received staging LD-CT scans for abdominal (n = 2) and urogenital (n = 3) cancer without radiological evidence of pulmonary metastases, respectively. According to our institutional guidelines, initial LD-CT was routinely performed in patients who qualified for chest imaging in the context of COVID symptoms. Follow-up CT scans were performed in patients with persistent or worsening symptoms after individual, inter-disciplinary consideration. The following parameters were collected retrospectively from the patient charts: age, gender, adiposity, diabetes mellitus, periphery artery disease, atrial fibrillation, heart failure, hypertension, hyperlipidemia, renal insufficiency, chronic obstructive pulmonary disease,history of nicotine abuse, COVID-19 therapy (steroid therapy, antiviral therapy and mechanical ventilation).

### 2.2 CT techniques and scanning protocol

All CT scans were conducted using a standardized institutional scanning protocol on a multidetector computed tomography scanner (iCT 256, Philips Healthcare, Best, The Netherlands). Scanning was performed during a full-inspiration breath-hold in craniocaudal direction from the thoracic inlet to the diaphragm. No contrast agent was applied. The following scan parameters were used: 29.4 ± 9.4 mAs, collimation 80 × 0.625 mm, pitch 0.763, tube voltage 120 kV, mean CTDIvol 2.5 ± 0.8 mGy, mean DLP 90.2 ± 29 mGy*cm.

### 2.3 Data post processing and image analysis

Images were interpreted using IMPAX EE (Agfa HealthCare N.V., Mortsel, Belgium). Two radiology residents (CN, EC) with at least five years of experience in diagnostic radiology reviewed the scans retrospectively in consensus using the source images. Both radiologists were blinded to the clinical data. Pulmonary involvement was quantified by a scoring system (max. 25 scoring points), which depends on the visual assessment of each lobe involved. In this score each lobe was awarded a CT score from 0 to 5, depending on the degree of inflammatory lung changes: score 0, 0% involvement; score 1, < 5% involvement; score 2, 5–25% involvement; score 3, 26–49% involvement; score 4, 50–75% involvement; and score 5, > 75% involvement. Depending on the sum score, severity of pulmonary involvement was categorized as mild (≤ 7 points), moderate (7–18 points) and severe (≥18 points) [13].

**Fig 2:**
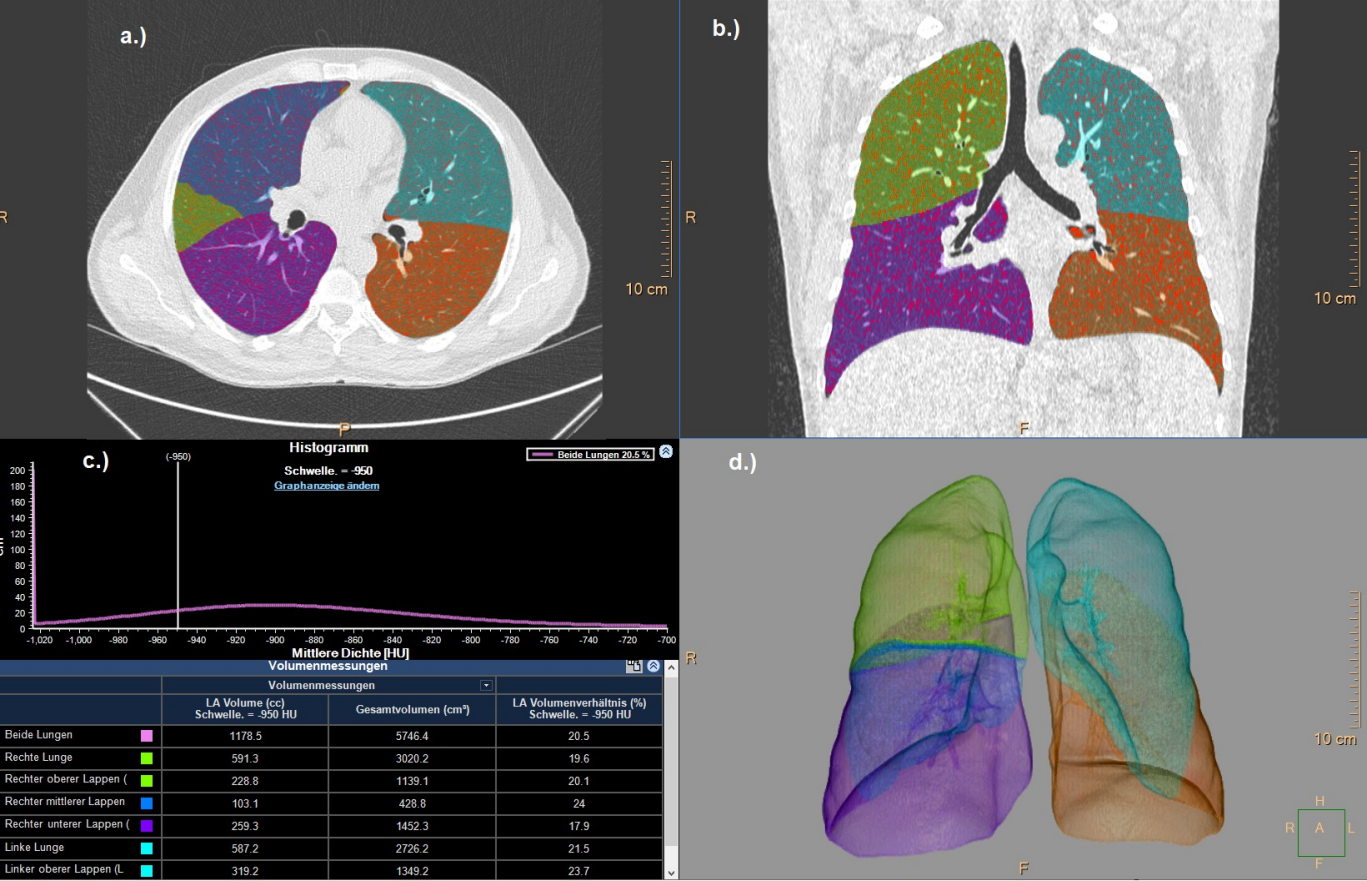
Automated lobe segmentation process and quantification of emphysema per lobe in a 45-year-old woman with COVID-19 pneumonia. (a./b./d.) Sequential segmentation of airways, lungs, and lobes. (c) Results of volumetric measurement of emphysema in both lungs, each lung, and each lobe (attenuation threshold of voxels, <-950 HU). Emphysematous voxels are shown in red. Reprinted under a CC BY license, with permission from Philips Healthcare, original copyright [2021].

Quantitative total lung and emphysema volume measurements were performed using a semi-automatic software (IntelliSpace Portal, Philips Healthcare, the Netherlands, Fig 2).

**Fig 3:**
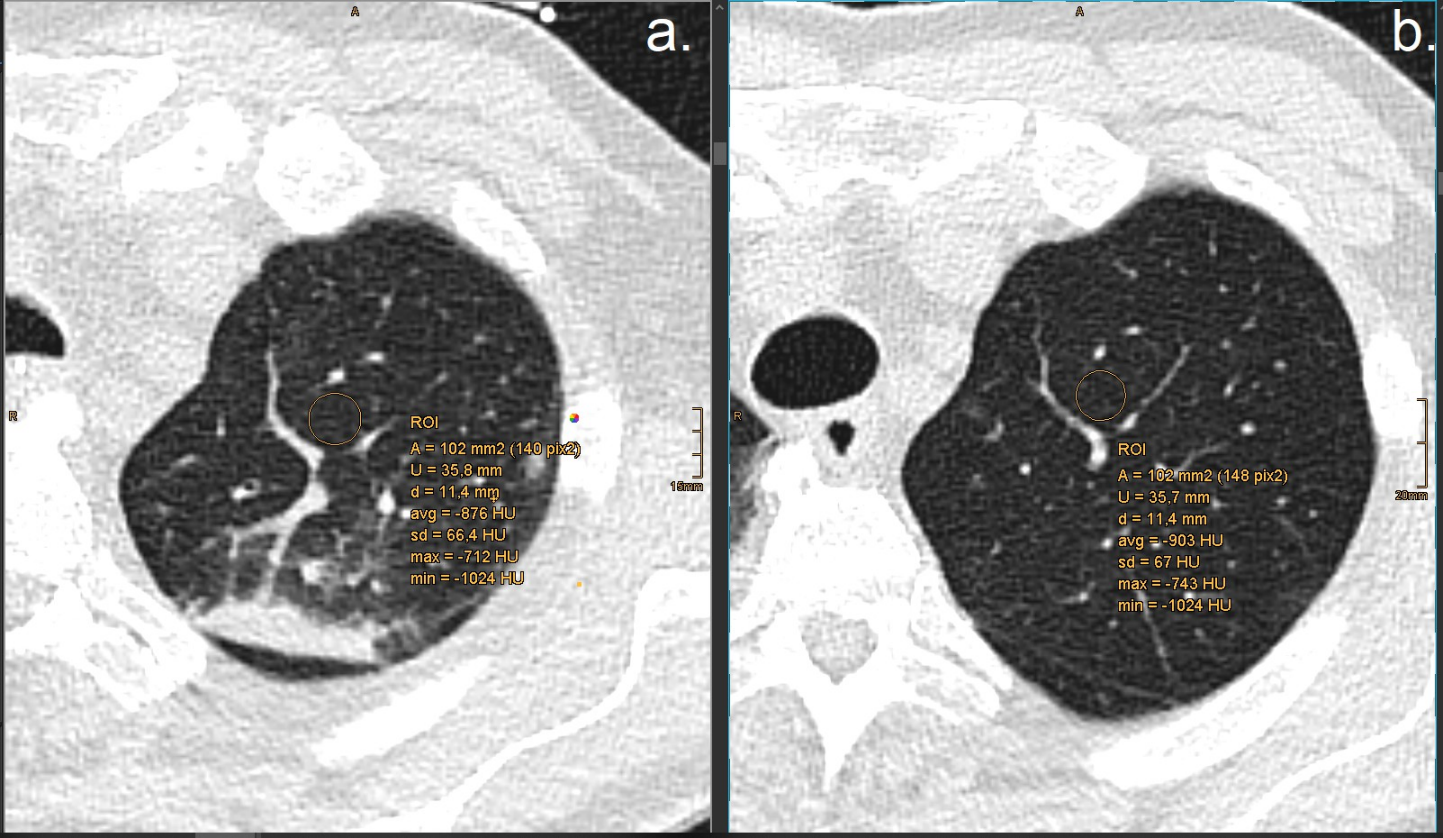
ROI measurements. Manual ROI measurements in the left upper lobe of a 45-year-old woman with COVID 19 pneumonia at initial CT scan (a.) and at follow-up CT scan after 45 days (b.), a decreasing in attenuation values by -27 HU can be seen between initial scan and follow-up.

The software automatically segmented the borders of the central airways and the segments of both lungs. Lungs were differentiated from the surrounding structures and automated lobe segmentation was performed. After segmentation, axial, coronal, sagittal and volume-rendered images were displayed. Automated lung segmentation was verified by the two readers and adjusted, if necessary. Voxels with an attenuation coefficient below -950 HU were considered as emphysematous [14,15]. The total lung volume, emphysema volume and percent ratio of these two parameters were automatically determined and recorded. After automated registration of the initial and the follow-up CT scan, circular ROIs were drawn centrally in both upper lobes, under the exclusion of opacifications and pulmonary vessels (Fig 3).

ROIs were automatically pasted to the post-COVID LD-CTs without specific user interaction. Attenuation values (in Hounsfield Units) were averaged and compared between CT scans.

### 2.4 Statistical analysis

Continous variables are presented as means with standard deviation or as median and range. Normal distribution was tested using the Shapiro-Wilk test. The paired Wilcoxon test was used to compare non-normally distributed data and the paired students t-test was used for normally distributed data. Categorical variables are reported as numbers and percentages and compared using the Chi-square or the Fisher Exact test, when appropriate. Statistical analysis was performed with R studio version 1.2.5033 (R Core Team 2013). A p-value of <0.05 was considered as statistically significant.

## 3. Results

A total of 185 COVID-19 patients received a LD-CT scan at the authors’ institution during the study period. Thereof, 32 patients (20 male; 12 female) met the inclusion criteria with at least one follow-up LD-CT. Demographic characteristics and quantitative measurements are summarized in Tables 1 and 2.

**Table 1:** Demographic characteristics of n = 32 patients with RT-PCR proven coronavirus disease.

Parameter	mean	sd	Count (n)	median	min	max	range
**Age (years)**	63.91	12.62		66	35	82	47
Gender*			m 20				
			f 12				
**CT follow up (days)**	52	65,53		26	4	259	255
**RT-PCR**			32				
Severity*							
**Mild, n (%)**			5 (16%)				
**Moderate, n (%)**			15 (47%)				
**Severe, n (%)**			12 (37%)				

RT-PCR–reverse transcription polymerase chain reaction, m–male, f–female, sd–standard deviation,.

**Table 2:** Demographic characteristics and quantitative measurements of n = 32 patients with RT-PCR proven coronavirus disease categorized by follow-up interval </> 30 days.

Parameter	pre	< 30 d (n = 19) post	p	pre	> 30 d (n = 13) post	p
**ROI r (HU), mean**	-869.37	-866.74	0.37	-842.54	-880.30	0.99
**ROI l (HU), mean**	-856.95	-879.47	0.93	-849.46	-875.84	0.99
**TLV (cc), mean**	3818.26	3700.89	0.94	3608.69	3708.15	0.68
**EV (cc), mean**	398.40	392	0.52	392.77	547.85	**0.045** *
**Ratio TLV/EV**	9.24	10.23	0.26	9.68	13.41	0.06
						**p**
**Diabetes Mellitus, n (%)**		9 (47%)			2 (15%)	0.14
**Adipositas, n (%)**		8 (42%)			1 (8%)	0.08
**Hypertension, n (%)**		14 (74%)			6 (46%)	0.23
**Hyperlipidaemia, n (%)**		7 (37%)			2 (15%)	0.35
**Coronary artery disease, n (%)**		4 (21%)			1 (8%)	0.59
**Atrial fibrillation, n (%)**		2 (11%)			1 (8%)	0.34
**Heart failure, n (%)**		4 (21%)			3 (23%)	1
**Periphery artery disease, n (%)**		5 (26%)			0 (0%)	0.13
**Renal insufficiency, n (%)**		5 (26%)			3 (23%)	1
**Ever smoker, n (%)**		5 (26%)			2 (15%)	0.76
**COPD, n (%)**		0 (0%)			1 (8%)	0.84

ROI–region of interest, TLV–total lung volume, EV–emphysema volume, COPD–chronic obstructive pulmonary disease, cc–cubic centimeters, d–days, n–count

*—statistically significant.

The mean patient age was 64 ± 13 years. On initial LD-CT, the severity of pulmonary involvement was categorized as mild in five cases (16%), moderate in 15 (47%) and severe in 12 cases (37%). On the initial CT, the mean total lung volume of the COVID 19-patients was 3733 ± 991 cc and the mean total emphysema volume was 396 ± 374 cc. In the control group of matched non-COVID patients, the mean total lung and emphysema volume was 4635 ± 1352 cc; 753 ± 536 cc at initial LD-CT scan and 4482 ± 1416 cc; 688 ± 469 cc at follow-up (mean 71 ± 52 days). The ratio of emphysema to total lung volume in this group did not differ significantly between initial scan as compared to baseline (p = 0.1), see Table 2.

**Fig 4:**
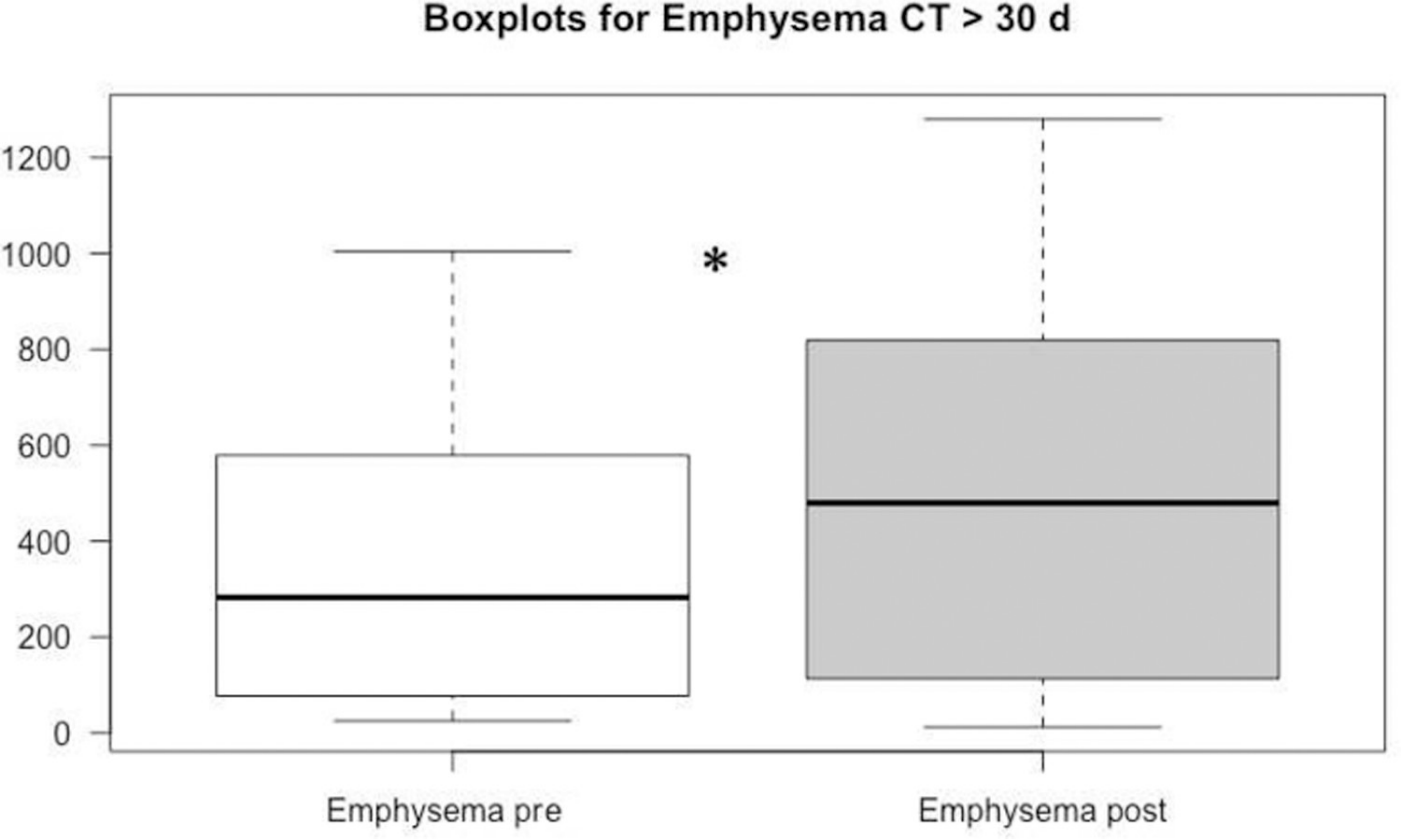
Boxplot for emphysema volume. Automated measured significantly higher amount of emphysema volume in follow-up CT scans >30 days as compared to the initial CT scan.

The mean follow-up period between the initial CT scan and the follow up scan was 52 ± 66 days (range: 5–259 days). The mean total lung and emphysema volume at follow-up was 3703 cc ± 937 and 455 cc ± 316, respectively. The ratio of emphysema to total lung volume at follow-up (11.5 ± 7.4) was not statistically different to the initial CT (p = 0.229). Among patients with a follow-up interval > 30 days, the total emphysema volume at follow-up (547.9 cc ± 417) was significantly larger than at baseline (392.7 cc ± 313, p = 0.045) and also significantly larger than the follow-up emphysema volume of patients with a follow-up interval < 30 days (392.8 cc ± 313; p = 0.045; see Fig 4).

**Fig 5:**
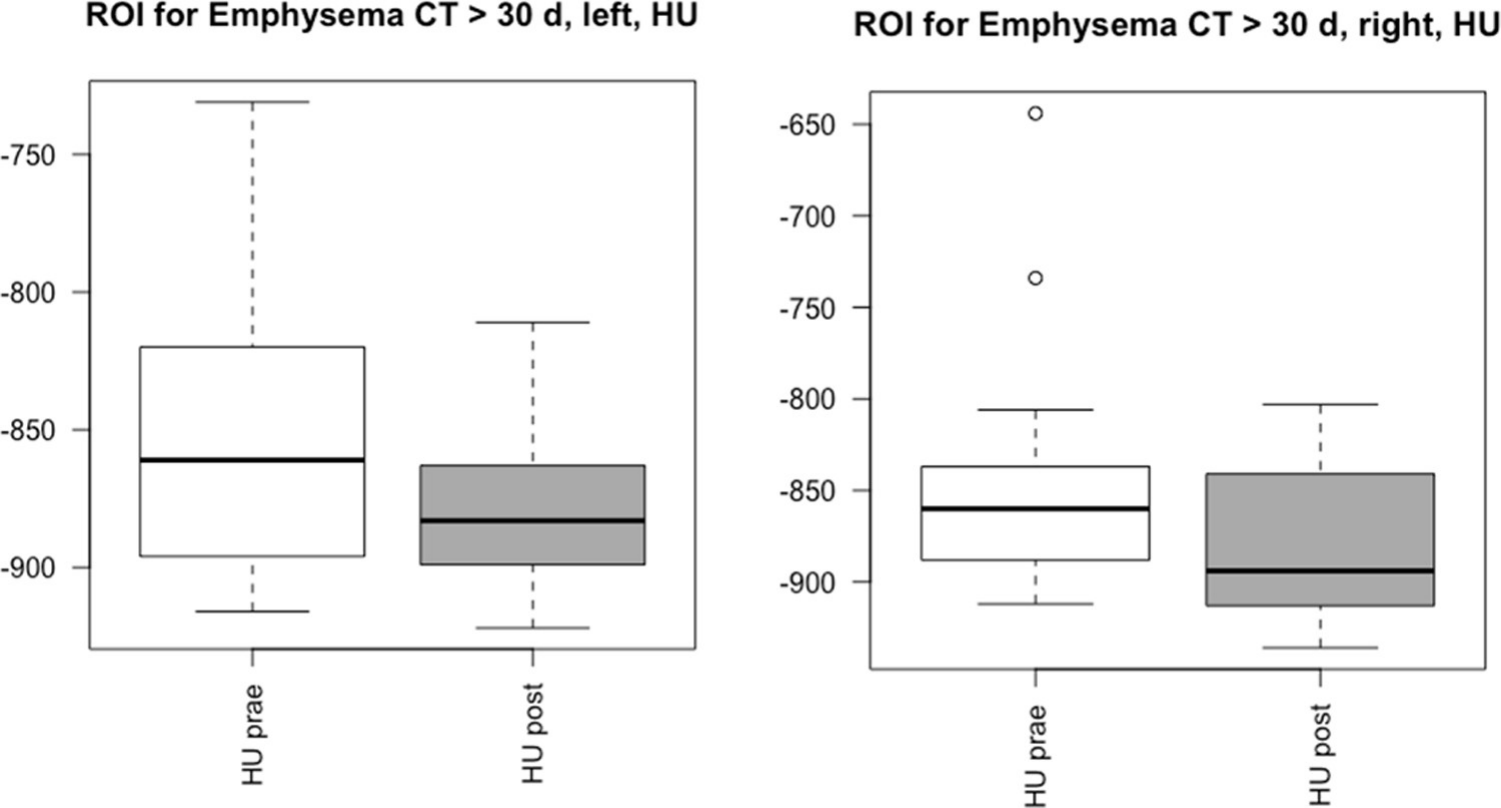
Boxplot for attenuation values. Manually measured attenuation values by ROIs in unaffected lung areas show no differences in the groups >30 days and < 30 days between initial CT scan (white) and follow-up CT scans (grey).

Moreover, in the subgroup > 30 days, the ratio of emphysema and total lung volume was by trend higher at follow-up (13.4 ± 10.0) than at initial CT (9.7 ± 6.4; p = 0.064). In the subgroup of patients with follow-up interval < 30 days (n = 13) the total emphysema volume was not significantly different between the initial CT scan (391.5 cc ± 421) and the follow-up CT scan (369.8 cc ± 180; p = 0,526). Patients’ characteristics did not differ significantly between the groups over/under 30 days follow-up (all p > 0.05), see Table 2. Manual ROI measurements of unaffected lung areas in the right and left upper lobe showed lower HU values, which did not yield statistical significance (p = 0.06, Fig 5).

Among 32 patients, 21 received steroid therapy (dexamethasone: 18, prednisolone: 3), 14 were treated by antiviral drug medication (remdesivir) and 18 received additional oxygen therapy. CT findings were not significantly different between the respective subgroups and the remaining population (each p > 0.05).

## 4. Discussion

The hypothesis of the current study was that inflammation-associated alveolar damage and vascular injury in COVID-19 patients causes emphysematous lung alterations. The semi-automatic quantitative results showed significantly increased emphysema volumes in patients with a follow-up > 30 days when compared to baseline (p = 0.04). Moreover, the ratio of emphysema-to-lung volume was increased in this subgroup by trend. Patients with a follow-up < 30 days showed no significant differences in the emphysema volume compared to baseline.

Previous studies have proposed semi-automated software applications to quantify COVID-19 pneumonia severity focusing on pulmonary opacification and emphysematous lung alterations [16–18]. In this context, pulmonary emphysema and COPD were identified as major risk factors for severe disease progression in patients with COVID-19. Grassi et. al showed that computer-aided pneumonia evaluation represents a reliable method to detect and to stratify inflammatory lung changes caused by COVID-19 according to severity [19,20]. Colombi et. al revealed that the amount of well-aerated lung parenchyma correlated positively with pulmonary function after infection [21]. To our knowledge, our study is the first that quantified the extent of emphysematous lung changes on follow-up LD-CT after COVID-19 infection. Besides noxious agents, infective lung disease represents a major risk factor for the development of emphysema. It is supposed that emphysema occurs as a consequence of damage to the airways distal to the terminal bronchioles [22]. Several autopsy studies revealed that diffuse alveolar damage and vascular injury are common pulmonary features in COVID-19 [8,9]. In a recently published study by Han et. al., fibrotic lung alterations were identified in more than one-third of patients six month after a severe COVID-19 infection [23]. Emphysema and pulmonary fibrosis both occur due to diffuse alveolar damaging [9], however, it remains unclear whether one entity affects the other or whether both develop independently [24,25]. The increase of emphysematous lung alterations may be significant for clinical routine, as it could deal as a surrogate parameter for the graduation of irreversible lung damage after COVID pneumonia. In the current study, patients with a follow-up ≥ 30 days had significant emphysematous lung alterations on CT when compared to baseline. In the subgroup of patients with <30 days of LD-CT follow-up, emphysematous lung changes were not significantly different. These patients are still within in the absorption stage that extends beyond 26 days from symptom onset. In this stage, pulmonary opacifications are still evident on LD-CT, which might mask emphysematous lung alterations on CT [5].

For internal control and validation, the volumetric measurements in the non-COVID control group did not differ significantly between initial scan and baseline. As another reference, ROIs were placed in unaffected areas of the lungs to determine whether a significant decrease of lung density could be observed in these areas as well. Our data indicate by trend minimal lower HU values at follow-up LD-CT scan, which supports our hypothesis. However, no significant decrease of attenuation in non-affected lung areas could be identified, thus it has to be assumed that alveolar damage with resulting emphysema increasement mainly occurs in visually affected lung areas, as a correlate of bronchoalveolar destruction and edema [9,24,25].

The typical clinical symptoms of lung emphysema are breathlessness, cough and chest pain. Conversely, up to 32% of COVID patients develop long-standing pulmonary symptoms such as fatigue, cough, chest tightness, breathlessness and palpitations, which is also known as the long COVID syndrome [26]. The pathogenesis of long COVID has not yet been elucidated in detail, however, recent studies suggest (1) virus-specific pathophysiologic changes; (2) immunologic aberrations and inflammatory damage in response to the acute infection and (3) expected sequelae of post-critical illness [27]. In this context, the development of inflammation-induced emphysematous lung alterations may contribute to impaired pulmonary function and thus long COVID. A limitation of the current study is that standardized assessment of pulmonary function at follow-up was not performed, e. g. pulmonary function tests, walk tests, and arterial blood gas assessments. Hence, our data do not reveal, if the observed emphysematous lung alterations translate into pulmonary symptoms or if they are an asymptomatic morphological phenomenon related to pulmonary remodeling after a COVID infection. Thus, larger further studies are warranted to reveal a correlation between post-COVID lung emphysema and impaired pulmonary function and to identify specific risk factors. Moreover, treatment recommendations and special aftercare programs need to be developed for patients with long-lasting post-COVID symptoms. These symptoms carry a high socioeconomic burden as they are associated with impaired working capacity, work losses and frequent use of medical services.

In addition to the retrospective, monocentric study design, the following limitations should be considered for clinical interpretation. First, the sample size was small, because follow-up CT was not regularly performed in favor of chest x-ray [28,29]. Moreover, pulmonary opacifications regress by time, hence, a reduced lung density at follow-up is not imperatively a result of pathological emphysematous remodeling but can display a well-ventilated lung parenchyma. However, we also observed emphysematous remodeling in areas without lung infiltrations at initial LD-CT. The semi-automated software did not quantify the pulmonary opacifications, however, this would not provide any additional information on the presence of preexisting emphysematous lung areas. Moreover, CT-morphological lung changes were not correlated with pulmonary functional tests, which impedes clinical interpretation of the presented results.

In conclusion, we demonstrated that semi-automatically determined emphysema volume was significantly increased in COVID-19 patients with a follow-up period >30 days. Whether these alterations are caused by emphysematous remodeling as a result of vascular or alveolar damage, or whether they represent a surrogate for reduced pulmonary opacifications, cannot be answered unambiguously at this point. However, we also observed emphysematous remodeling in areas without lung infiltrations at LD-CT during acute infection. Thus, it has to be assumed, that it might be a combination of both factors. Further studies with longer observation periods would be needed to verify our preliminary results and to fully clarify the clinical impact of COVID-19 on lung emphysema development.

## Supporting information

S1 Data COV data. (XLSX)

## Abbreviations

CC: cubic centimeters
CT: computed tomography
CI: confidence interval
COPD: chronic obstructive pulmonary disease
EV: emphysema volume
GGOs: Ground-glass opacities
HU: Hounsfield unit
LD-CT: low-dose computed tomography
kV: kilovolt
mAs: milliampere-seconds
MRI: magnetic resonance imaging
PACS: picture archiving and communication system
ROI: region of interest
RT-PCR: reverse-transcription polymerase chain reaction
SD: standard deviation
TLV: total lung volume
